# A Mild Juvenile Onset Canavan Disease With Atypical Clinical Presentation and MRI Brain Features

**DOI:** 10.1002/jmd2.70031

**Published:** 2025-07-11

**Authors:** Preeya Rehsi, Ata Siddiqui, Rahul Singh, Obioma Ihezue, Rebecca Halligan

**Affiliations:** ^1^ Department of Inherited Metabolic Diseases Evelina London Children's Hospital London UK; ^2^ Department of Inherited Metabolic Disease Great Ormond Street Hospital London UK; ^3^ Department of Neuroradiology Evelina London Children's Hospital London UK; ^4^ Department of Children's Neurosciences Unit Evelina London Children's Hospital London UK; ^5^ Department of Paediatrics East Surrey Hospital Redhill UK

**Keywords:** atypical presentation, Canavan disease, intention tremor, neuroimaging, whole genome sequencing

## Abstract

This case report highlights an atypical presentation of Canavan disease (CD) in a 13‐year‐old female with intention tremor and fine motor difficulties. Neuroimaging revealed symmetrical changes in various brain regions initially suggesting a neurometabolic or mitochondrial disorder. However, further investigations, including biochemical analysis and whole genome sequencing, confirmed a diagnosis of CD. Unlike classical presentations, this case exhibited milder symptoms and unusual MRI findings, contributing to the expanding clinical and radiological spectrum of CD. The importance of recognizing such atypical presentations is emphasized for accurate diagnosis and management of CD.

**Summary**

- Milder cases of Canavan disease can present with atypical neuroimaging features, broadening the clinical and radiological phenotype.

## 1 Case Report

**FIGURE 1:**
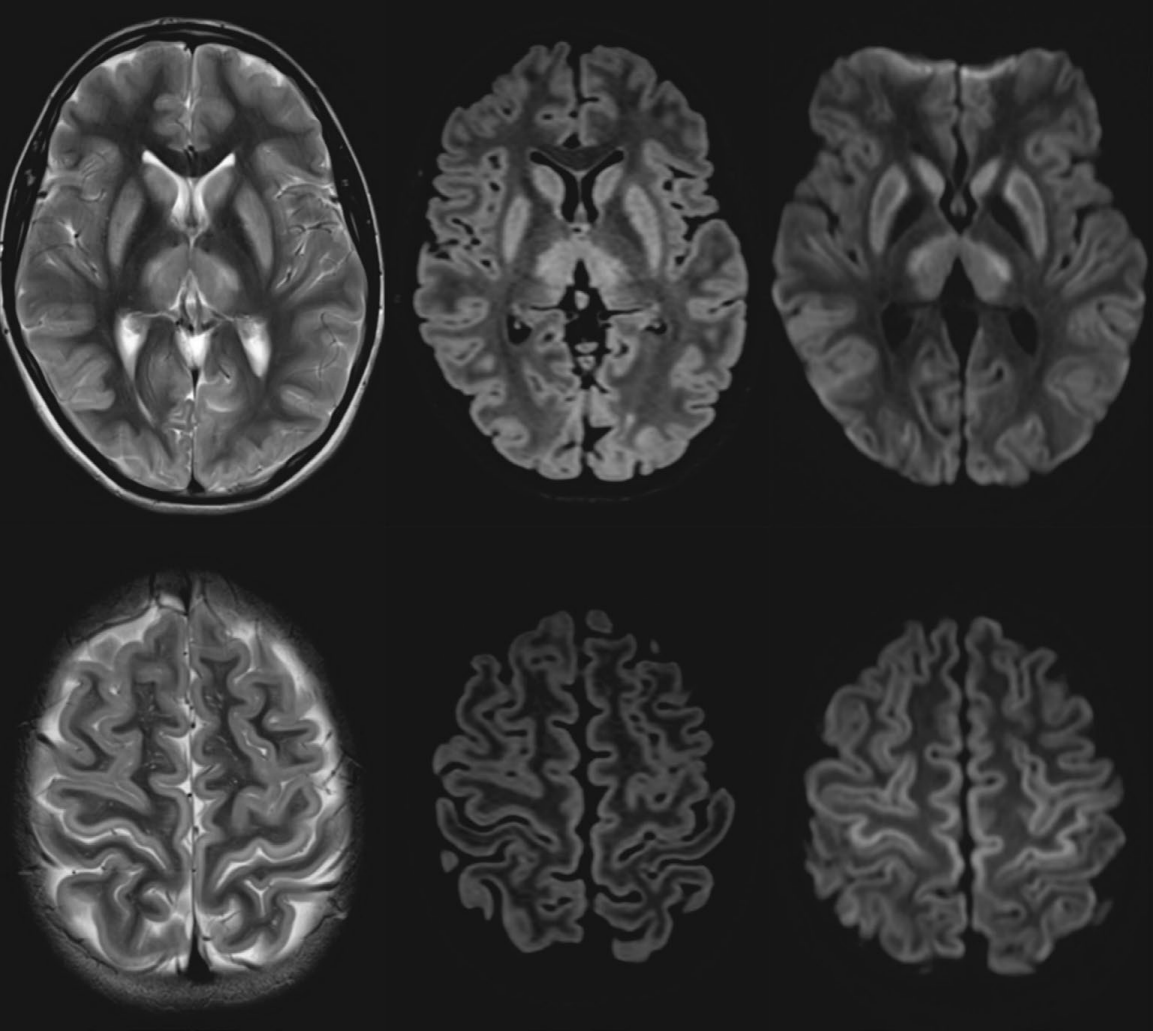
MRI with axial T2W, FLAIR and DWI images taken at the level of the basal ganglia (top row) and towards the vertex (bottom row) showing bilateral symmetrical basal ganglia, thalamic and cortical signal changes. The cortical changes are more evident within the precentral gyri (primary motor cortex). Subtle early subcortical white matter involvement is seen in the occipital lobes.

A well 13‐year‐old female presented with a 5‐year history of intention tremor and difficulty with fine motor skills. MRI brain revealed symmetrical changes in the putamen, thalami, caudate nuclei and occipital cortex bilaterally, raising the possibility a neurometabolic or mitochondrial disorder (Figure 1). Biochemical investigations revealed a normal lactate, acylcarnitine profile and plasma amino acids. Urine organic acid analysis revealed mildly elevated N‐acetyl aspartic acid (NAA). Trio whole genome sequencing confirmed homozygosity for the *ASPA* c.820G>A p.(Gly274Arg) likely pathogenic variant, consistent with a diagnosis of Canavan disease (CD).

**FIGURE 2:**
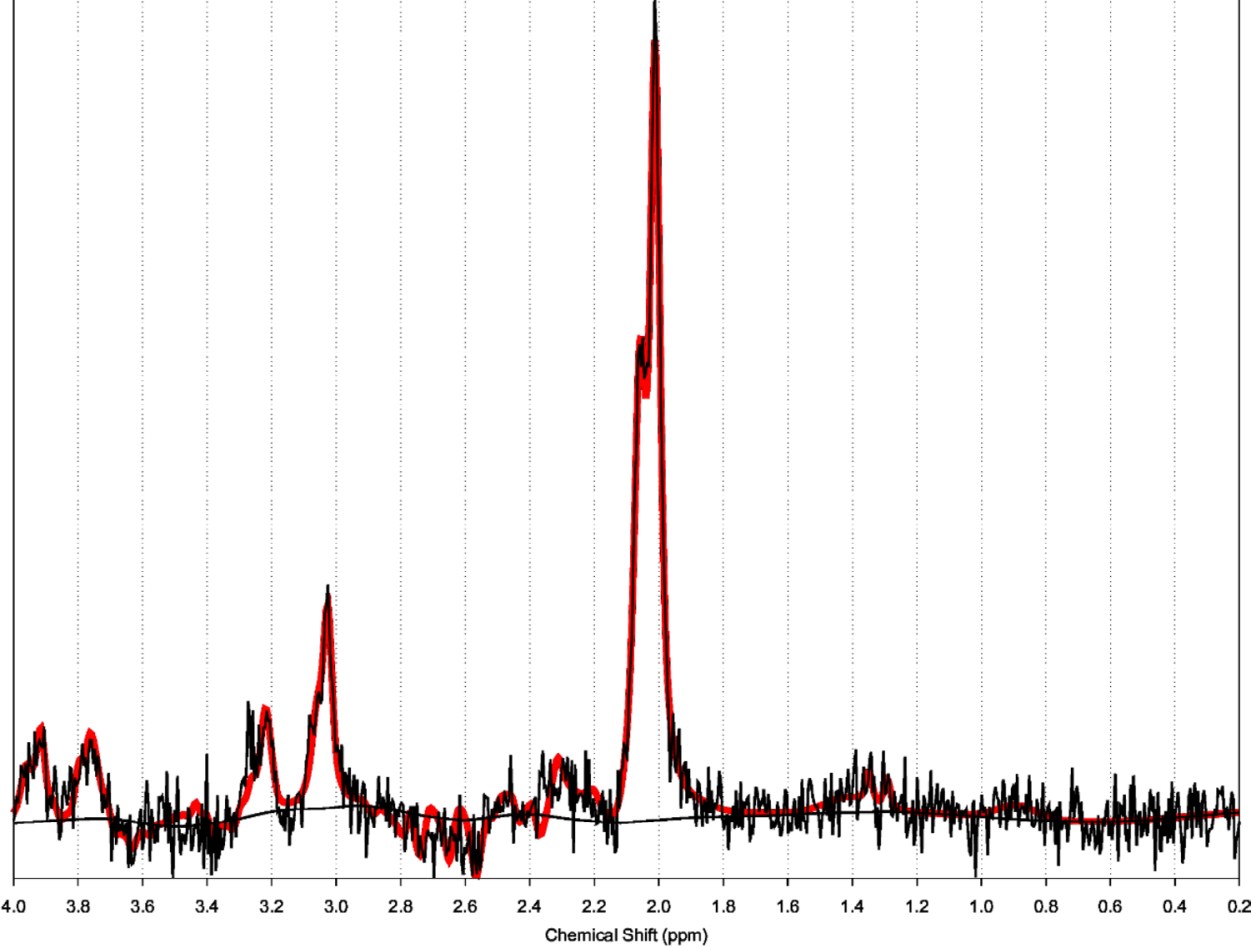
Single voxel MR spectroscopy at TE 288 ms taken from the basal ganglia shows a significantly elevated NAA peak.

After 12 months of follow up there is no symptom progression. However, she has developed new onset Type 1 Diabetes Mellitus with classical symptoms of polyuria, polydipsia, weight loss, and mild diabetic ketoacidosis at presentation. An interval MRI brain with spectroscopy showed stable appearances with an elevated NAA peak on MRS (Figure 2). In hindsight, there was early subcortical white matter involvement in the occipital lobes.

## 2 Discussion

CD is classically a life‐limiting, neurodegenerative condition characterized by infantile onset severe neurodevelopmental delay followed by arrest, seizures, visual impairment, and death in early infancy [1]. There is, however, emerging evidence of clinical heterogeneity associated with *ASPA* variants [2].

This G274R variant found in our patient was first described by Shaag et al. in 1995 in the homozygous state, but presenting with a severe phenotype including axial hypotonia from 3 months of age with white matter changes on MRI brain [3]. The same variant was found in a Pakistani family with moderate to severe presentation of CD however their neuroimaging has not been described [4].

Subsequent reports of milder phenotypes related to the G274R variant have been described. A child of Greek origin developed seizures at 11 weeks of life but continued to show developmental progress and by 4 years of age is walking with support and speaking single words. T2 weighted imaging showed increased signals in the periphery of the thalami, putamina, head of the caudate nucleus, and subcortical white matter, as well as the tegmentum of the pons and the white matter around the 4th ventricle [2]. This patient had two separate homozygous variants, one being the G274R and the other K213E. The latter variant has been proven to express wildtype protein activity. Our case is comparable to a patient reported in Turkey in 2020 with a similar clinical symptom of an intention tremor presenting at 13 years of age. MRI brain showed symmetric hyperintensities isolated to the pons and this patient was homozygous for the G274R variant [5].

Classically on MRI, CD affects the white matter in a diffuse and symmetrical pattern, with bilateral involvement of the globus pallidus and less marked involvement of the cerebellum, thalami, and brainstem. There is diffusion restriction without contrast enhancement in the affected white matter [6]. A spongiform degeneration is classically described, separating CD from other leukodystrophies. The imaging findings in our patient are atypical, and our case contributes to the broadening clinical and radiological phenotype in CD, demonstrating the emergence of milder cases.

The interindividual variability seen among patients with the G274R variant and other variants may be attributable to unidentified epigenetic and environmental factors, and further research is required to explore this.

## Author Contributions

As first author P.R. contributed to the design, acquisition of data, and drafting of the work. Co‐authors A.S. and R.S. provided interpretation and expertise in the neuroimaging aspects of the work. Co‐author O.I. participated in providing clinical detail. Senior author and guarantor R.H. participated in the conception, design, acquisition of data, critical review and revision of the work. All authors reviewed the work and provided final approval of the version to be published.

## Ethics Statement

The authors have nothing to report.

## Consent

All procedures followed were in accordance with the ethical standards of the responsible committee on human experimentation (institutional and national) and with the Helsinki Declaration of 1975, as revised in 2000 (5). Informed consent was obtained from the patient for being included in this report.

## Conflicts of Interest

The authors declare no conflicts of interest.

## Data Availability

The authors do not have authorization from the families to share personal data. Sharing anonymized data can be discussed upon request to Dr. Preeya Rehsi.
